# Pals1 functions in redundancy with SMAP1 to inhibit Arf6 in order to prevent Rac1-dependent colorectal cancer cell migration and invasion

**DOI:** 10.1038/s41417-022-00570-2

**Published:** 2022-12-09

**Authors:** Julia Harms, Simona Mareike Lüttgenau, Christin Emming, Justine Guske, Katrin Weber, Thomas Wagner, Larissa Schowe, Pavel Nedvetsky, Michael P. Krahn

**Affiliations:** Medical Clinic D, Medical Cell Biology, University Hospital of Münster, Münster, Germany

**Keywords:** Colorectal cancer, Cell biology, Gene expression

## Abstract

Downregulation of cell–cell adhesion and increased motility are prerequisites for the metastasis of cancer cells. We have recently shown that downregulation of the tight junction adapter protein Pals1 in colorectal cancer cells results in an increase of cell migration, invasion, and metastasis due to the enhanced activation of Arf6 and Rac1. We now reveal a redundancy between the Arf6-GAP SMAP1 and Pals1 in regulating Arf6 activity and thereby Rac1-dependent cell migration. The gene encoding SMAP1 is frequently disrupted in microsatellite instable colorectal cancer specimen and cell lines. In cells expressing SMAP1, deletion of Pals1 leads to disturbed formation of tight junctions but has no impact on Arf6 activity and cell migration. In contrast, inactivation of both SMAP1 and Pals1 results in enhanced Arf6/Rac1 activity and increased cell migration and invasion. Furthermore, analyzing patient cohorts, we found a significant decrease in patient’s survival when both genes were downregulated, in contrast to cases, when expression of only one of both genes was affected. Taken together, we identified a redundancy between SMAP1 and Pals1 in the regulation of activation of Arf6/Rac1, thereby controlling cell migration, invasion, and metastasis of colorectal cancer cells.

## Introduction

Despite recent advances in prevention, early diagnosis and therapy, colorectal cancer remains to be one of the most frequent cancer types and a major cause of tumor-related deaths. One crucial hallmark of the life-threatening colorectal cancer progression is reprogramming and dedifferentiation of epithelial cells, reflected by a downregulation of cell–cell and cell–matrix adhesion contacts. This process finally leads to an epithelial-to-mesenchymal transition (EMT) or at least a transient acquisition of mesenchymal characteristics and the disintegration of single cells from the solid tumor and their subsequent dissemination. Concomitantly with the downregulation of cell–cell adhesion contacts, apical-basal polarity is lost and consequently several polarity regulators have been identified as tumor suppressors [1].

In epithelia, apical-basal polarity is established and maintained by a tightly regulated balance between two apical complexes (the PAR/aPKC- and the Crumbs complex) and their basolateral counterparts, the Discs large/Lethal (2) giant larvae/Scribble module and the kinases Par1 and LKB1 [2]. Within the Crumbs (Crb) complex, the adaptor protein Pals1 (Protein associated with Lin-7 One) stabilizes the transmembrane protein Crb (Crb3 in most classical epithelia) and links it to the myosin regulator PATJ (Pals1-associated TJ protein) [3–6]. Loss of the Crb complex results in disturbed or delayed formation of tight junctions (TJ) in cultured cells [7–12] and Crb3-knockout mice display microvilli defects and disturbed determination of the apical membrane in the intestine and other epithelial tissues [13, 14].

Crb3 is downregulated in cells overexpressing Snail or ZEB1, transcription factors which are capable to induce EMT [15–17]. Conversely, the knockdown of Crb3 enhances the expression of Snail upon TGFβ-stimulation [18]. In a mouse tumor model, Crb3 is downregulated upon depletion of p53 and Rb, resulting in enhanced EMT [7]. Thus, Crb3 seems to be a major gatekeeper preventing EMT. Apart from its role in cell polarity and cell–cell adhesion, Crb and Pals1 have been found to be upstream regulators of the Hippo pathway, thus controlling cell proliferation, organ size and stem cell maintenance in *Drosophila* and mammals [18–25].

Cell migration and invasion of tumor cells is driven by the formation of lamellipodia and filopodia at the leading edge, which are formed and destructed by a dynamic rearrangement of the actin cytoskeleton. One of the key factors in this process is the small GTPase Rac1, which increases actin filament elongation and branching by activating the WAVE complex, which enhances Arp2/3 complex activity [26]. Rac1 itself is activated by various guanine exchange factors (GEFs), including Tiam1, which links Rac1 to the apical polarity regulator PAR3 [27]. Increased activation of Rac1 results in enhanced cell migration and invasion. Consequently, Rac1 or its GEFs are upregulated or mutated in several types of cancer, including colorectal cancer, correlating with a poor prognosis for the patients [28].

Apart from its regulation via GEFs and GAPs (GTPase activating proteins), Rac1 can also be activated indirectly by another small GTPase, ADP-ribosylation factor 6 (Arf6) [29–34]. Arf6 is implicated in several cellular processes, including vesicle trafficking, endocytosis and modulation of the actin cytoskeleton [35]. In addition to Rac1 activation, Arf6 facilitates the recycling of Rac1 to the plasma membrane from endosomes [36, 37]. Outside of the leading edge, stable lamellipodia formation is inhibited by decreasing Rac1 activity at α4-Integrin/Paxillin complexes, which recruit the GIT1-family of Arf GAPs to inhibit Arf6 activity [38]. Arf6 has also been described to recruit and activate Arf1 to the plasma membrane during *Salmonella* infection, resulting in enhanced WAVE-dependent actin polymerization and lamellipodia formation [39]. Arf6 is frequently overexpressed in several types of cancer, including invasive breast cancer, melanoma and pancreatic cancer and Arf6 activity is driving these tumor cells to become more invasive [34, 40–45].

In a previous study [46], we showed that Pals1 binds and inhibits Arf6 in colorectal cancer cells. Deletion of Pals1 results in enhanced activation of Arf6, which in turn activates Rac1, leading to increased cell migration, invasion and metastasis. However, we now found that deletion of Pals1 in other colorectal cancer cell lines did not result in enhanced Arf6/Rac1-dependent cell migration. Investigating the discrepancy between these results, we found Pals1 to function in redundancy with the Arf6-GAP SMAP1 to control Arf6 activation. SMAP1 is frequently deleted in microsatellite instable colorectal cancer specimen and cell lines [47]. Deletion of Pals1 in SMAP1-deficient cells or inactivation of both genes disinhibits Arf6 resulting in an increase Rac1-dependent cell migration and invasion.

## Materials and methods

### Cell culture

HCT116/HCT116ΔPals1 were cultured in DMEM high glucose (4.5 g/L, Sigma-Aldrich) and Caco-2/Caco-2ΔPals1 and RKO/RKOΔPals1 were cultured in DMEM low glucose (1 g/L Sigma-Aldrich), DLD1 and -derived lines and SW48 were cultured in RPMI-1640 (Sigma-Aldrich) medium supplemented with 10% FCS and 1% antibiotics (streptomycin/penicillin). All cell lines were cultivated at 37 °C under 5% CO_2_ atmosphere and passaged every 3–4 days. Transfections were performed with Lipofectamin^TM^ 2000 (Thermo Fisher Scientific) or Metafectene® (Biontex) according to the manufacturer’s introductions.

To establish stable knockout cells lines by using CRISPR/Cas9, cells were transfected with the following guides together with Cas9 from px459: Pals1: GCCCTGGAGATTTGGGCACC; SMAP1: GTATCTGTTCTGCTGTCCAT. Non transfected cells were eliminated with 36 h puromycin selection and subsequently single-cell-clones were generated and analyzed for efficient knockout by western blot and sequencing. For all cell lines, a second independent guide (Pals1: ATTAGCCGGATAGTAAAAGG, SMAP1: GTTATCTGTTTTCAGAGCAG) was analyzed, showing the same results.

### Immunofluorescence analysis

Cells were grown on coverslips and fixed with 4% PFA in phosphate buffer pH 7.4 for 7 min or with methanol at −20 °C for 10 min. After washing three times with PBS cells were incubated for 1 h with PBS + 2.5% horse serum and 0.05% Saponin (PBSS) or 0.1% Triton X-100 (PBST). Subsequently, primary antibodies diluted in the same solution were added for 2 h at RT or overnight at 4 °C. After washing three times with PBST/PBSS the coverslips were incubated with the secondary antibody (diluted 1:1000 in PBSS + HS/PBST + HS), DAPI (1:1000, Invitrogen Life Technologies) for 1 h. Finally, coverslips were washed with PBS and mounted in mowiol. The following primary antibodies were used: mouse anti-Pals1 (1:100, Santa Cruz #365411), rabbit anti-Pals1 (1:1000, Proteintech #17710-1AP), rabbit anti-PATJ (1:100, raised in this study), mouse anti-PATJ (1:50, DSHB Hybridoma Product AFFN-INADL-1-3G6), rabbit anti-ZO-1 (1:100, Cell Signaling #13663), rabbit anti-E-Cadherin (1:100, Cell Signaling #3195), mouse anti-E-Cadherin (1:100, Santa Cruz #21791), rat anti-E-Cadherin (1:100, Santa Cruz #59778), guinea pig anti-Crb3a (1:200, raised in this study), mouse anti-Occludin (1:100, Santa Cruz #271842).

### Cell lysis and Western blotting

Cell lysates were made with Laemmli buffer (for Western blotting) or with TNT-buffer (pulldown experiments; 150 mM NaCl, 50 mM Tris, 1 mM MgCl_2_, 1 mM CaCl_2_, 1% Triton X-100, pH 7.5 + protease inhibitor cocktail). SDS-PAGE and Western blotting were performed according to standard procedures. The following primary antibodies were used: mouse anti-Pals1 (1:500, Santa Cruz #365411), rabbit anti-Pals1 (1:1000, Proteintech 17710-1AP), mouse anti-ß-Actin (1:500, Santa Cruz #47778), mouse anti-Rac (1:500, Santa Cruz #514583), rabbit anti-α-Actinin 4 (1:1000, Cell Signaling #6487), mouse anti-α-Actinin 4 (1:2000, Santa Cruz #17829), mouse anti-Arf6 (1:500, Santa Cruz #7971), rabbit anti-E-Cadherin (1:1000, Santa Cruz #7870), mouse anti-SMAP1 (1:1000, Abnova H00060682-B01P).

### Wound healing assay

Cells in 12-wells were separated by culture-Inserts (2 well, ibidi) until confluency. Assays were started with the removal of the insert and pictures were taken by a ZEISS Observer Z1. Data were analyzed using ImageJ.

### Transwell matrigel invasion assay

Thincert cell culture inserts (8 µm, Greiner Bio-One) were coated with Cultrex® basement membrane extract (BME, final concentration 1 mg/ml, diluted in respective culture medium). After incubation at 37 °C for 2 h, 1.5 ml of DMEM with 10% FCS were added to the lower compartment. A total of 100,000 cells in serum-free medium were added to the upper compartment and incubated at 37 °C. Subsequently remaining cells in the upper compartment were removed and cells in the lower compartment were stained with crystal violet after PFA fixation. For quantification, cells were removed from the membrane with methanol and OD at 570 nm was measured with the microplate reader infinite® M200 (Tecan Trading AG).

### G-LISA

Cells were cultured in 10 cm dishes until confluency. The Rac1 G-LISA activity assay (#BK128, Cytoskeleton Inc.) was performed according to manufacturer’s instructions, using 0.5 mg/ml protein concentration of cell lysates. The results of the G-LISA were obtained by OD 490 measurement with the microplate reader infinite® M200 (Tecan Trading AG).

### Rac1 activity quantification by FRET-based biosensor

Cells were seeded on 18-well slides (ibidi µ-Slide 18 well) pre-coated with a basal membrane extract (1 mg/ml laminin, 1 mg/ml fibronectin, 0,9 mg/ml collagen IV) and transfected with the Rac1 biosensor (pTriEx4-Rac1-2G, gift from Olivier Pertz, Addgene plasmid # 66110) [48]. As controls donor-only (mTFP1) and acceptor-only (Venus)-transfected cells were used. Cells were grown for 2–3 days after transfection and analysis was done with a confocal microscope (Leica TCS SP8) using the FRET Sensitized Emission Wizard (LAS X software).

### Statistical analysis

All data is presented as mean ± SEM of at least three independent experiments. Statistical significance was evaluated with unpaired t-test or One-way ANOVA using GraphPad Prism: ns > 0.05, *p < 0.05, **p < 0.01, ***p < 0.001.

## Results

### Pals1 depletion results in enhanced cell migration and invasion in HCT116 cells

**Fig. 1:**
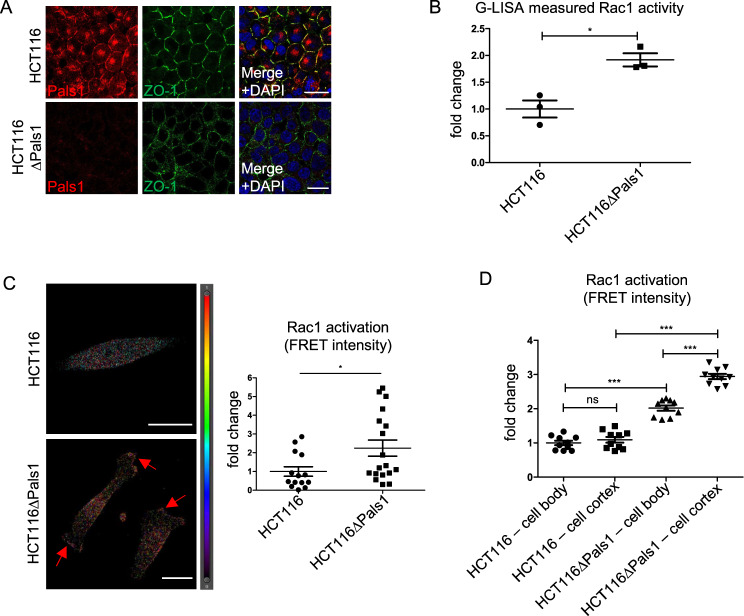
Loss of Pals1 in a colorectal cancer cell line HCT116 results in TJ defects, enhanced migration and invasion. **A** Immunostaining of confluent HCT116 and HCT116ΔPals1 cells with indicated antibodies, **B** Activation of Rac1 in wild type and Pals1-deficient HCT116 was quantified using G-LISA assay. **C** Representative images and quantification of the FRET signal of a biosensor targeting active Rac1, transfected in HCT116 and HCT116ΔPals1 cells. Results are representative of 4 experiments. **D** Quantification of Rac1 biosensor FRET signals at the cell body and the cell cortex in HCT116 and HCT116ΔPals1 cells. Scale bars are 20 µm in **A**, **C**.

As previously reported [46], deletion of Pals1 in the colorectal cancer cell line HCT116 results in disturbed tight junction formation as evaluated by staining for ZO-1 (Fig. 1A) and Occludin (Supplementary Fig. 1A), a loss of its canonical interaction partners PATJ and Crb3a from cell–cell contacts (Supplementary Fig. 1A, B), enhanced collective cell migration (Supplementary Fig. 1C) and increased invasion through extracellular matrix (Supplementary Fig. 1D). Mechanistically, we confirmed an enhanced activation of Arf6 by GTPase pulldown assay (Supplementary Fig. 1E), which results in an increased activation of Rac1 (quantified using G-LISA assays and a FRET-based biosensor [48], Fig. 1B, C). Notably, the increase in Rac1 activation in Pals1-deficient cells is higher at the cell cortex compared to the cell body (Fig. 1D).

### Knockout of Pals1 does not enhance cell motility in other colorectal cancer cell lines

**Fig. 2:**
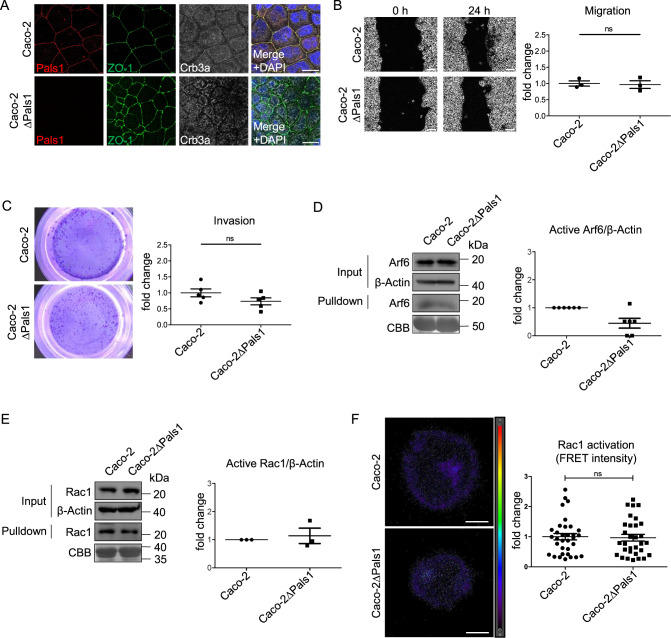
Deletion of Pals1 in Caco-2 cells does not result in enhanced migration and invasion or upregulation of active Arf6 or Rac1. **A** Immunostaining of confluent Caco-2 and Caco-2ΔPals1 cells with the indicated antibodies. **B** Representative images from wound healing assays of Caco-2 and Caco-2ΔPals1 cells and the corresponding quantification (*N* = 3). **C** Representative images and quantification of transwell matrigel invasions assays of Caco-2 and Caco-2ΔPals1 cells (*N* = 5). **D** Western blot and CBB-stained gel of pulldown experiments to detect active Arf6 from lysates of Caco-2 and Caco-2ΔPals1 cells (*N* = 6). **E** Western blot and CBB-stained gel of pulldown experiments to detect active Rac1 from lysates of Caco-2 and Caco-2ΔPals1 cells (*N* = 3). **F** Representative images and quantification of the FRET signal of a biosensor targeting active Rac1, transfected in Caco-2 and Caco-2ΔPals1 cells. Results are representative of 4 experiments. Scale bars are 20 µm in A and F, 100 µm in B.

Tumors often display a remarkable heterogeneity, which is also reflected by tumor-derived cell lines. To elucidate whether our findings regarding the effects of Pals1 loss are valid for other colorectal cancer cell lines, we established a Pals1 knockout in another cell line, Caco-2. In line with the literature, knockout of Pals1 resulted in loss of Crb3a from cell-cell contacts (Fig. 2A). However, in contrast to Pals1-deficient HCT116 cells, cortical localization of the TJ markers ZO-1 and Occludin is only slightly affected, but cells appeared smaller and irregularly shaped within a confluent monolayer (Fig. 2A and Supplementary Fig. 2A). As in HCT116 cells, PATJ was lost from cell-cell contacts (Supplementary Fig. 2B). Surprisingly, the two-dimensional cell migration of the Pals1-deficient Caco-2 cells was unchanged and their invasion was rather decreased compared to the wildtype control line (Fig. 2B, C). Furthermore, Rac1 and Arf6 activities in Pals1-depleted Caco-2 cells were either unaffected or even lower than in the parenteral cell line (Fig. 2D–F).

**Fig. 3:**
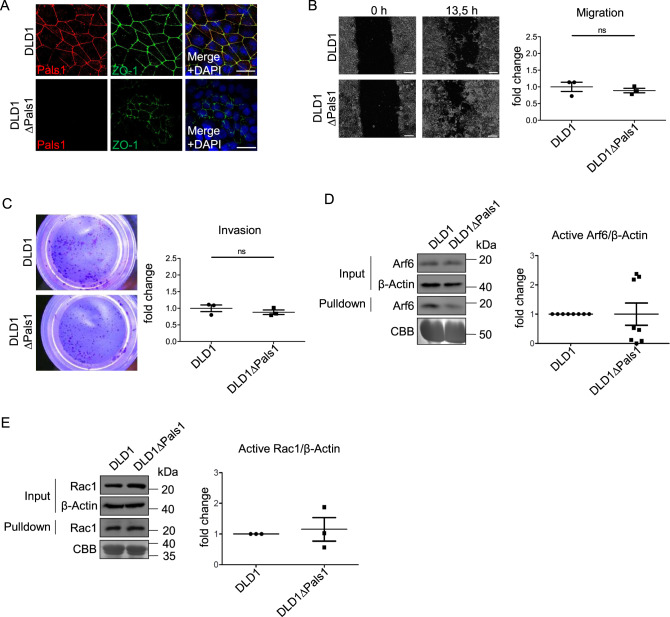
Pals1-deficient DLD1 do not exhibit increased Arf6/Rac1 activity or enhanced cell migration/invasion. **A** Immunostaining of confluent DLD1 and DLD1ΔPals1 cells with the indicated antibodies. **B** Representative images from wound healing assays of DLD1 and DLD1ΔPals1 cells and the corresponding quantification (*N* = 3). **C** Representative images and quantification of transwell matrigel invasion assays of DLD1 and DLD1ΔPals1 cells (*N* = 3). **D** Western blot and CBB-stained gel of pulldown experiments to detect active Arf6 from cell lysates of DLD1 and DLD1ΔPals1 cells (*N* = 8). **E** Western blot and CBB-stained gel of pulldown experiments to detect active Rac1 from cell lysates of DLD1 and DLD1ΔPals1 cells (*N* = 3). Scale bars are 20 µm in A and 100 µm in B.

In contrast to HCT116, Caco-2 cells retain a high degree of epithelial polarization and bear no KRAS and PIK3CA mutations, whereas HCT116 cells are mutant for these oncogenes. Therefore, we checked a third cell line, DLD1, which is mutant for KRAS and PIK3CA similarly to HCT116 and less differentiated than Caco-2. Similarly to HCT116 cells, Pals1-deficient DLD1 cells displayed a reduction of cortical ZO-1 and Occludin, suggesting defects in TJ formation, as well as irregular cell shapes (Fig. 3A and Supplementary Fig. 3A). However, regarding their motility, Pals1-depleted DLD1 cells behaved similarly Caco-2∆Pals1 cells and showed no enhanced but rather a slight decrease in cell migration and invasion (Fig. 3B, C). This is in line with the observation that Arf6- (Fig. 3D) and Rac1-activation (Fig. 3E) was not increased in DLD1∆Pals1 cells in comparison to control cells.

**Fig. 4:**
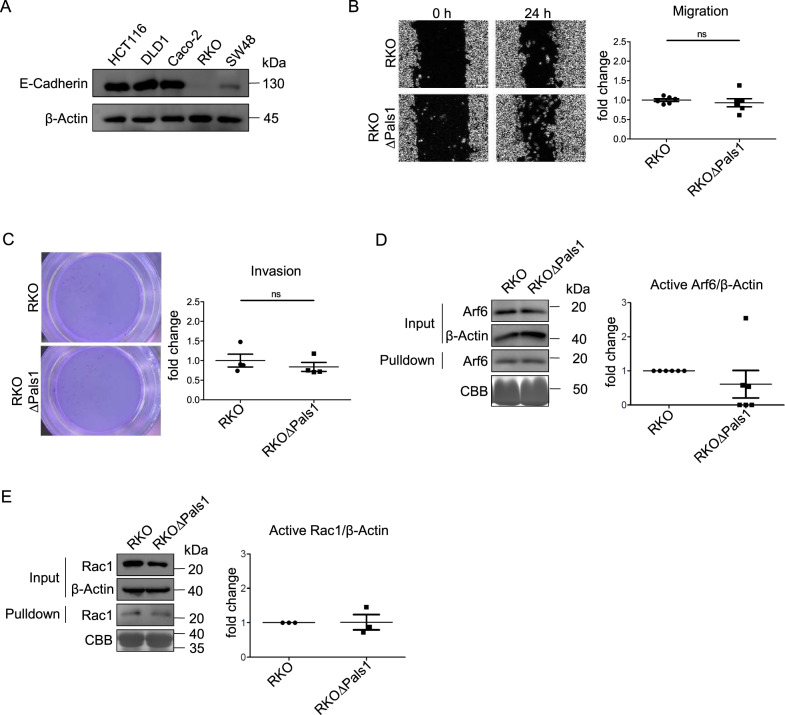
Knockout of Pals1 in mesenchymal-like RKO cells does not affect cell motility. **A** Western blot analysis of the expression of E-Cadherin in different colorectal cancer cell lines. **B** Representative images from wound healing assays of RKO and RKOΔPals1 cells and the corresponding quantification (*N* = 6). **C** Representative images and quantification of transwell matrigel invasion assays of RKO and RKOΔPals1 cells (*N* = 4). **D** Western blot and CBB-stained gel of pulldown experiments to detect active Arf6 from cell lysates of RKO and RKOΔPals1 cells (*N* = 6). **E** Western blot and CBB-stained gel of pulldown experiments to detect active Rac1 from cell lysates of RKO and RKOΔPals1 cells (*N* = 3). Scale bars are 100 µm in B.

We reasoned that HCT116 might be less differentiated than Caco-2 and DLD1, which was supported by the notion, that TJ are weaker and sometimes discontinuous in HCT116 cells but are more robust in DLD1 and Caco-2 cells. Therefore, we repeated our experiments in RKO cells, which do not express detectable levels of E-Cad (Fig. 4A) and seem to form no cell-cell contacts at all (data not shown). However, even in this highly de-differentiated cell line, deletion of Pals1 did not enhance cell motility (Fig. 4B, C). Again, levels of activated Rac1 and Arf6 did not increase upon Pals1 knockout in RKO cells (Fig. 4D, E). Thus, in contrast to Caco-2, DLD1 and RKO, HCT116 cells seem to be sensitized for Pals1 depletion in the context of Arf6 activity.

### Pals1 controls Arf6 activity in redundancy with the Arf6-GAP SMAP1

**Fig. 5:**
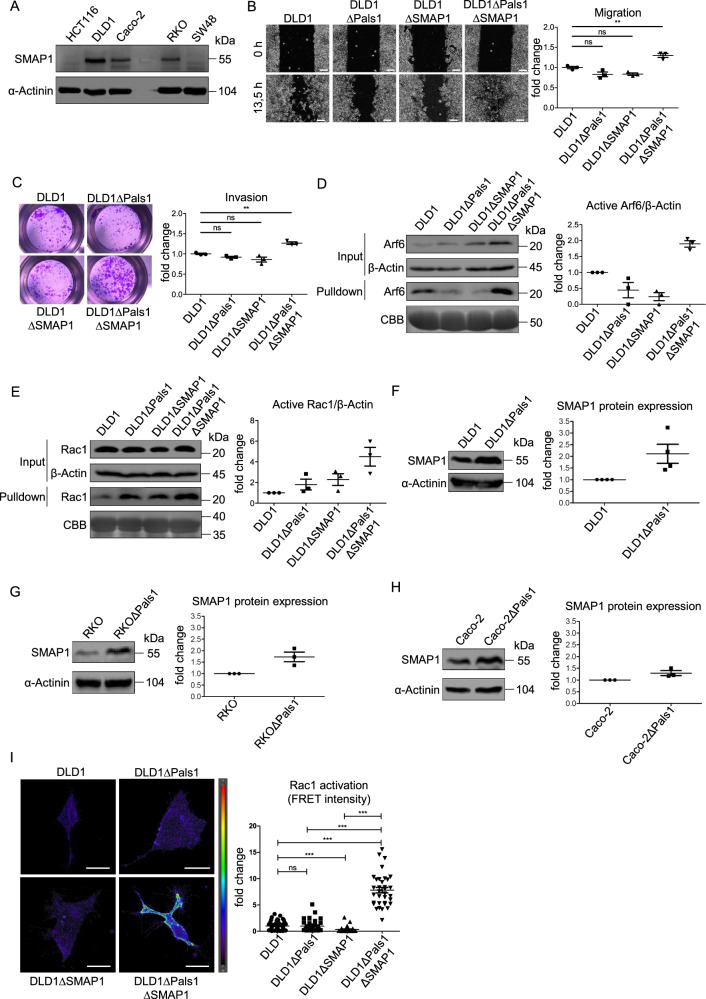
Pals1 and SMAP1 function in redundancy to control Arf6-dependent Rac1 activation and cell migration. **A** Western blot analysis of the expression of SMAP1 in different colorectal cancer cell lines. **B** Representative images from wound healing assays of DLD1, DLD1ΔPals1, DLD1ΔSMAP1 and DLD1ΔPals1ΔSMAP1 cells and the corresponding quantification (*N* = 3). **C** Representative images and quantification of transwell matrigel invasion assays of DLD1 and DLD1ΔPals1 cells (*N* = 3). **D** Western blot and CBB-stained gel of pulldown experiments to detect active Arf6 from cell lysates of DLD1, DLD1ΔPals1, DLD1ΔSMAP1 and DLD1ΔPals1ΔSMAP1 cells (*N* = 3). **E** Western blot and CBB-stained gel of pulldown experiments to detect active Rac1 from cell lysates of DLD1, DLD1ΔPals1, DLD1ΔSMAP1 and DLD1ΔPals1ΔSMAP1 cells (*N* = 3). Note that in the Rac1 blot of the output an empty lane was cutted out between DLD1∆Pals1 and DLD∆SMAP1 cells. **F**–**H** Western blot and quantification of SMAP1 expression in wild type and Pals1-deficient DLD1 **F**, RKO **G**, and Caco2 **H** cells (*N* > = 3). **I** Representative images and quantification of the FRET signal of a biosensor targeting active Rac1, transfected in indicated DLD1 cell lines. Results are representative of 4 experiments. Scale bars are 100 µm in B and 20 µm in **I**.

One possibility to explain the different migration behavior of HCT116 and other colorectal cancer cell lines would be that in HCT116 cells, Arf6 is more sensitive towards the regulatory function of Pals1. Notably, Sangar et al. reported that in microsatellite instable colorectal cancer specimen and cell lines, the expression of the Arf6-GAP SMAP1 is frequently disturbed due to deletions or insertions in an adenine repeat, resulting in a premature stop codon [47]. The authors also demonstrated that HCT116 cells carry a homozygous truncation of SMAP1, whereas RKO are heterozygous and DLD1 are wildtype for SMAP1. We confirmed that Caco-2, DLD1 and RKO cells express robust levels of SMAP1, whereas no full-length protein but only faint degradation products were seen in HCT116 cells (Fig. 5A).

Strikingly, deletion of SMAP1 in DLD1∆Pals1 and RKO∆Pals1 cells recapitulates the findings in HCT116 cells: Enhanced cell migration and invasion (Fig. 5B, C and Supplementary Fig. 4A, B) as well as increased Arf6 and Rac1 activity (Fig. 5D, E and I and Supplementary Fig. 4C). In contrast, deletion of SMAP1 alone in DLD1 or RKO cells rather decreases cell motility (Fig. 5B, C and Supplementary Fig. 4A, B). Of note, SMAP1 is upregulated in Pals1-deficient DLD1 and RKO cells, whereas in Caco-2∆Pals1 cells, SMAP1 expression in only slightly increased (Fig. 5F–H), indicating a compensatory mechanism upon knockout of Pals1.

**Fig. 6:**
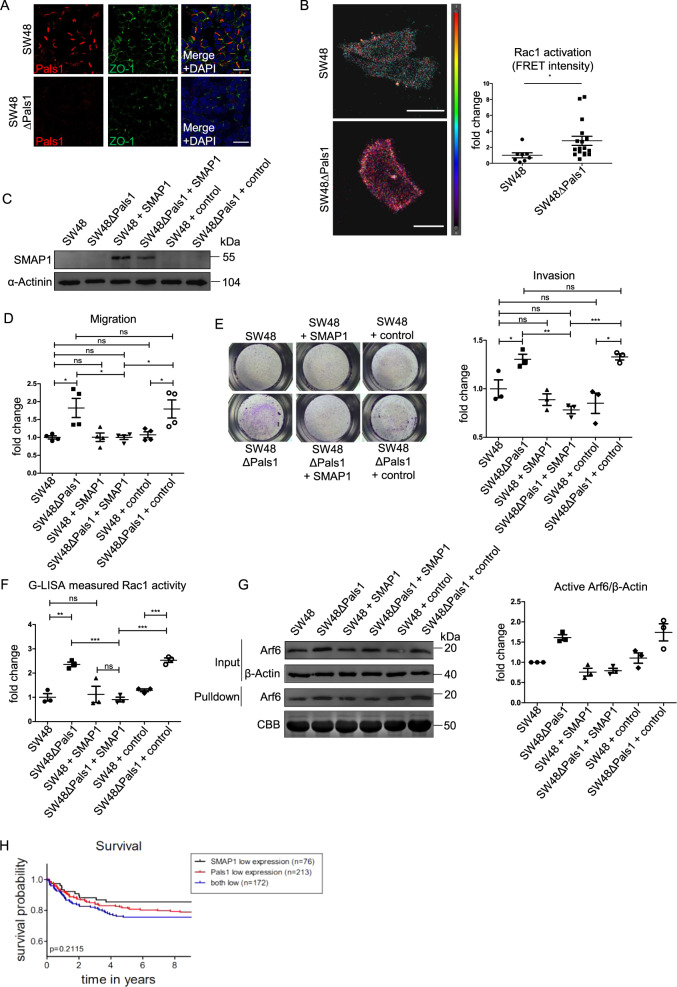
SW48∆Pals1 cells display enhanced Arf6/Rac1 activation and increased cell migration, which can be rescued by SMAP1 transfection. **A** Immunostaining of confluent SW48 and SW48ΔPals1 cells with the indicated antibodies. **B** Representative images and quantification of the FRET signal of a biosensor targeting active Rac1, transfected in SW48 and SW48ΔPals1 cells. Results are representative of 3 experiments. **C** Western blot of cell lines with and without SMAP1 overexpression. Empty vector was used as negative control. **D**, **E** Quantification of cell migration (scratch assay, **D**) and invasions assay (**E**) of the indicated cell lines. **F** Rac1 activation of the indicated cell lines quantified by G-LISA. **G** Western blot and CBB-stained gel of pulldown experiments to detect active Arf6 from cell lysates of the indicated cell lines (*N* = 3). **H** Survival probability of colorectal cancer patients with only low Pals1 expression, only low SMAP1 expression or low Pals1 and low SMAP1 expression. Scale bars are 20 µm in **A** and **B**.

In a parallel approach, we tested another SMAP1-deficient colorectal cancer cell line (Fig. 5A), SW48 and observed indeed a similar behavior as with HCT116 cells: Deletion of Pals1 further reduced the cortical TJ marker ZO1 (Fig. 6A) and displaced PATJ and Crb3a from the junctions (Supplementary Fig. 5A). Moreover, Pals1-deficient SW48 cell exhibited an enhanced cell migration (Supplementary Fig. 5B) and increased levels of active Arf6 and Rac1 (Fig. 6B and Supplementary Fig. 5B, C).

To further support our hypothesis that SMAP1 and Pals1 function in redundancy in regulating Arf6 activation, we re-expressed SMAP1 in wild type and Pals1-deficient SW48 cells (Fig. 6C). Indeed, re-expression of SMAP1 reduced migration and invasion of Pals1-deficient SW48 cells to levels comparable to the wild type cell line (Fig. 6D, E). Moreover, the enhanced Arf6 and Rac1 activation in Pals1-deficient cells was reduced to control levels when SMAP1 was re-expressed (Fig. 6F–G). Thus, cells which express either SMAP1 or Pals1 displayed a reduced migratory capacity and only cells lacking both proteins exhibit an increased activation of Arf6/Rac1 and migrated/invaded faster.

Taken together, we concluded from these experiments that Pals1 functions in redundancy with SMAP1 to control the levels of active Arf6 and thus Rac1-mediated cell migration in colorectal cancer cells.

### Downregulation of Pals1 and SMAP1 correlates with poor prognosis in colorectal cancer

Finally, we checked whether the proposed mechanism of Pals1 controlling tumor cell migration/metastasis in redundancy with SMAP1 is also reflected in patient cohorts. Indeed, the overall survival analysis shows a decreased survival of patients with downregulation of both Pals1 and SMAP1 in comparison to those cases, where only one of them was downregulated (Fig. 6H).

## Discussion

Invasive cancer cells forming metastases in distant organs are the main cause of tumor-related deaths. Thus, understanding of the molecular pathomechanisms leading to enhanced invasion and metastasis is one of the most important prerequisites for development of new therapies.

Recently, we revealed a new function of the polarity regulator Pals1 in controlling cell motility of colorectal cancer cells by inhibiting Arf6, which in its turn controls Rac1-dependent lamellipodia formation and tumor cell migration/invasion [46]. Notably, rescue experiments with Pals1-variants lacking the domains responsible for the interaction with its canonical complex partners Crb or PATJ, suggest that Pals1 functions in this context independently of these proteins, although deletion of Pals1 in all cell lines results in displacement of PATJ from cell–cell contacts.

A role for Pals1 in TJ formation and apical-basal polarity has already been reported before—downregulation of Pals1 has been shown to result in delayed formation of TJ [10], disturbed trafficking of E-Cad [49] and impaired contact inhibition in cultured mammalian cells [18]. The latter fact can be explained by the increased activity of the transcriptional co-activator YAP in cells with decreased Pals1 expression, resulting in enhanced cell proliferation, as has been demonstrated in cultured MDCK cells and murine kidneys, which consequently develop a cystic phenotype [25]. All these functions are assigned to Pals1 as a part of the Crb complex, as deletion of Crb in mice or downregulation of Crb or PATJ in cultured cells results in similar phenotypes [8, 11, 13, 14, 23, 50, 51].

In HCT116 cells, deletion of Pals1 enhances collective cell migration but also renders isolated HCT116 cells more motile without any effects on cell–cell contacts or assembled TJ. Mechanistically, we confirmed that Pals1 interacts with Arf6 independently of its GTP/GDP-bound state and the level of active Arf6 is increased upon depletion of Pals1 in HCT116 cells due to an enhanced transcription and translation/stability of Arf6 [46]. GTP-bound Arf6 has been shown to localize to lamellipodia of migrating cells and overexpression of constitutively active Arf6 is sufficient to enhance lamellipodia formation and cell migration [52, 53]. However, all studies reporting an effect of Arf6 binding partners on cell migration documented a modulation of Arf6 activity without changes in total Arf6 levels. By contrast, deletion of Pals1 in HCT116 cells does not affect the ratio of active to total Arf6. Nonetheless, due to increased total Arf6 levels in Pals1-deficient cells, the amount of active Arf6 is enhanced, too. Consequently, active Arf6 enhances Rac1-dependent cell migration and invasion, which can be counterbalanced by inhibiting Arf6 or Rac1 in Pals1-deficient cells [46]. The colocalization of endogenous Pals1 with Arf6 at lamellipodia but not at cell-cell contacts in migrating cells and the fact that Arf6 (and Rac1) are strongly enriched at lamellipodia in Pals1-deficient cells suggests that Pals1 inhibits Arf6 in order to control cell migration [46].

Notably, deletion of Pals1 in three other colorectal cancer cell lines (Caco-2, DLD1 and RKO) alone does not increase cell migration or Arf6/Rac1 activity, due to the expression of the Arf6-specific GAP SMAP1. SMAP1 is frequently mutated in microsatellite instable colorectal cancer specimen and cell lines [47]. HCT116 is one of the lines with biallelic mutant SMAP1, whereas Caco-2, DLD1 and RKO are either wild type or heterozygous for mutant SMAP1, which explains the different phenotypes upon Pals1 knockout in these cell lines. However, co-deletion of Pals1 and SMAP1 but not of SMAP1 alone in DLD1 or RKO cells enhances Arf6/Rac1 activity as well as cell migration and invasion of these cell lines. Vice versa, re-expression of SMAP1 in SW48∆Pals1 cells, which lack functional SMAP1, rescues migration and invasion behavior and decreased Arf6/Rac1 activation. In contrast to DLD1 cells, which develop proper TJ and express high levels of E-Cadherin, RKO cells lack E-Cad expression and thus do not assemble AJ or TJ. The fact that both cell lines behave similarly upon deletion of Pals1 and SMAP1 further supports our hypothesis that the role of Pals1 in controlling Arf6 is independent of its known junctional function, e.g., positioning ZO-1 at the TJ. Although we observed changes in the accumulation of TJ proteins in Pals1-deficient HCT116 and DLD1 cells, Caco2 cells did not display significant TJ defects. Further experiments are needed to address the question, whether deletion of Pals1 and SMAP1 result in changes in the formation and dynamic of TJ during cell polarization in different cell lines and whether this might contribute to an enhanced migratory phenotype.

Interestingly, ubiquitous deletion of SMAP1 in mice alters vesicle trafficking in erythroid-myeloid progenitor cells, resulting in myelodysplasia. Occasionally, mice also developed myeloid leukemia, whereas no other tumorous phenotypes are reported [54]. Thus, deletion of SMAP1 seems to affect Arf6-dependent processes only in a specialized subset of otherwise wild type, non-transformed cells. This might be to some extent due to other Arf6-specific GAPs with overlapping functions.

Finally, evaluation of mRNA expression of colorectal cancer specimen (TCGA project) revealed a poorer survival prognosis of patients suffering from colorectal cancer if tumor samples display low Pals1 and SMAP1 expression in contrast to tumors with either Pals1 or SMAP1 downregulation. These data indicate that the mechanism revealed in this study likely contributes to the pathogenesis of colorectal cancer in patients. Moreover, inhibition of Arf6 reduces cell migration of Pals1/SMAP1-double deficient cancer cells and might be considered as a future therapeutic approach for a subset of colorectal cancer patients.

## Supplementary information

Supplementary Figures 1-6

## Data Availability

The data generated in this study are available within the article and its supplementary data files.
