# Supplementary material for: Pals1 functions in redundancy with SMAP1 to inhibit Arf6 in order to prevent Rac1-dependent colorectal cancer cell migration and invasion
Source: Cancer Gene Ther. 2022 Dec 9;30(3):497–506. doi: 10.1038/s41417-022-00570-2 (PMC10014575; doi:10.1038/s41417-022-00570-2)

Supplementary Figure 1

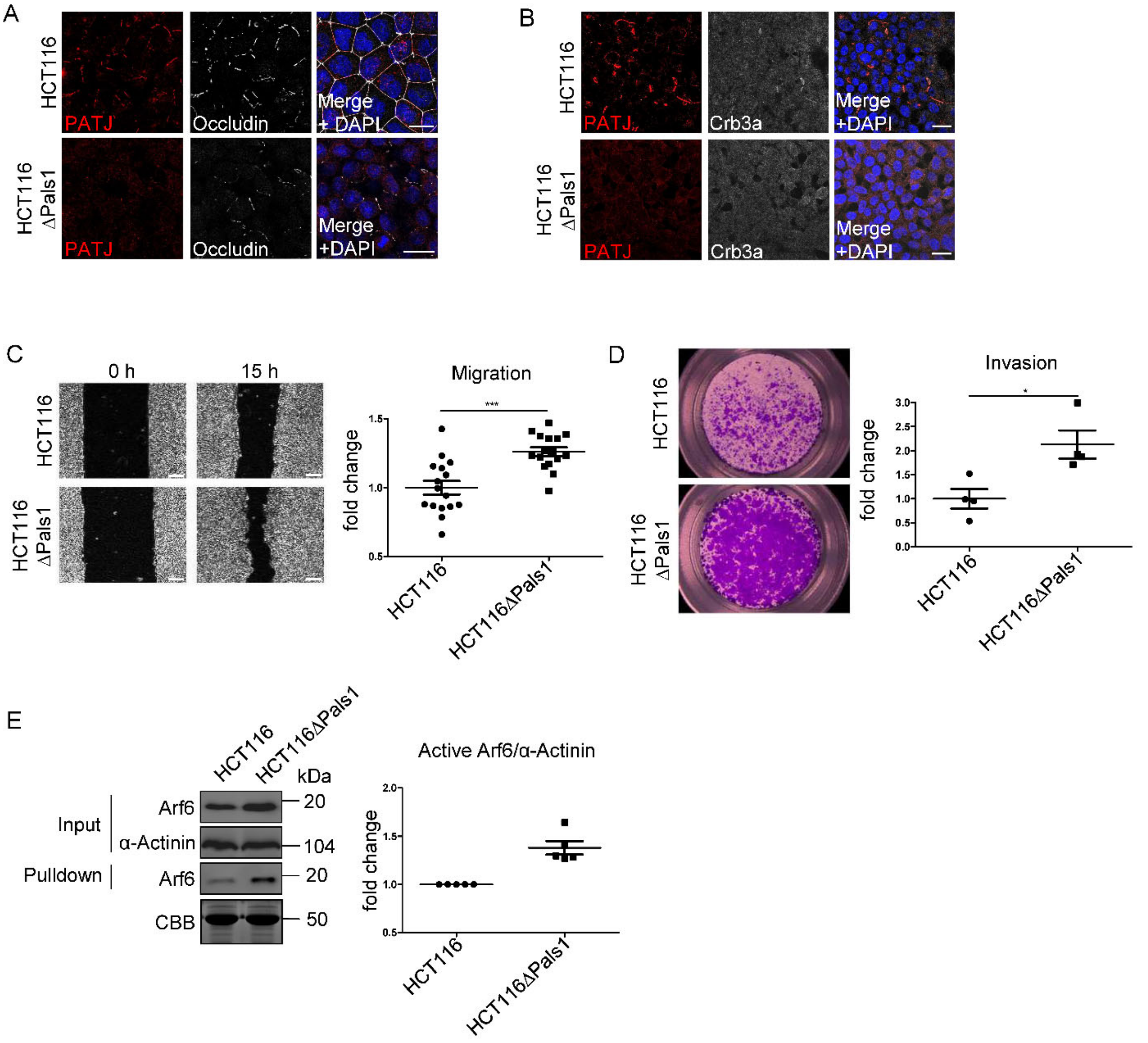

Supplementary Fig. 1: Pals1-depletion in HCT116 cells results in enhanced cell migration
A-B Immunostaining of confluent wild type or Pals1-deficient HCT116 cells with the indicated antibodies. **C** Representative images from wound healing assays of HCT116 and HCT116ΔPals1 cells and the corresponding quantification (*N* = 16). **D** Representative images and quantification of transwell matrigel invasion assays of HCT116 and HCT116ΔPals1 cells (*N* = 4). **E** Western blot and quantification of pulldown experiments for active Arf6 in HCT116 and HCT116ΔPals1 cells, using the recombinant effector protein GGA3 (Golgi-localized γ-adaptin ear-containing, Arf-binding protein 3) fused to GST. GST-GGA3 is visualized by Coomassie brilliant blue (CBB) staining (*N* = 5). Scale bars are 20 µm in A and B, 100 µm in C.

Supplementary Figure 2

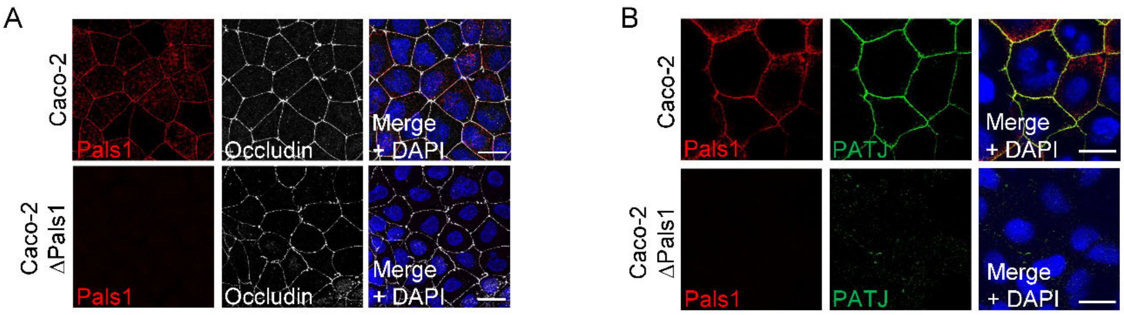

Supplementary Fig. 2: Occludin and PATJ stainings in Pals1-deficient Caco-2 cells

A-B Immunostaining of confluent wild type or Pals1-deficient Caco-2 cells with the indicated antibodies. Scale bars are 20 μm.

Supplementary Figure 3

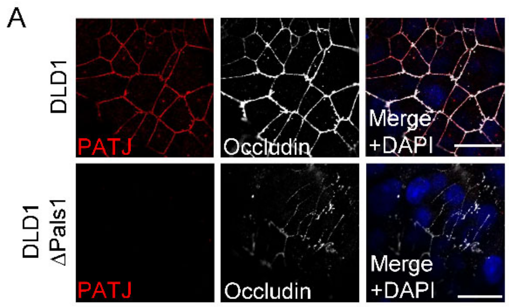

Supplementary Fig. 3: Occludin and PATJ are displaced in Pals1-deficient DLD1 cells

A Immunostaining of confluent wild type or Pals1-deficient Caco-2 cells with antibodies against Occludin and PATJ. Scale bars are 20 µm.

Supplementary Figure 4

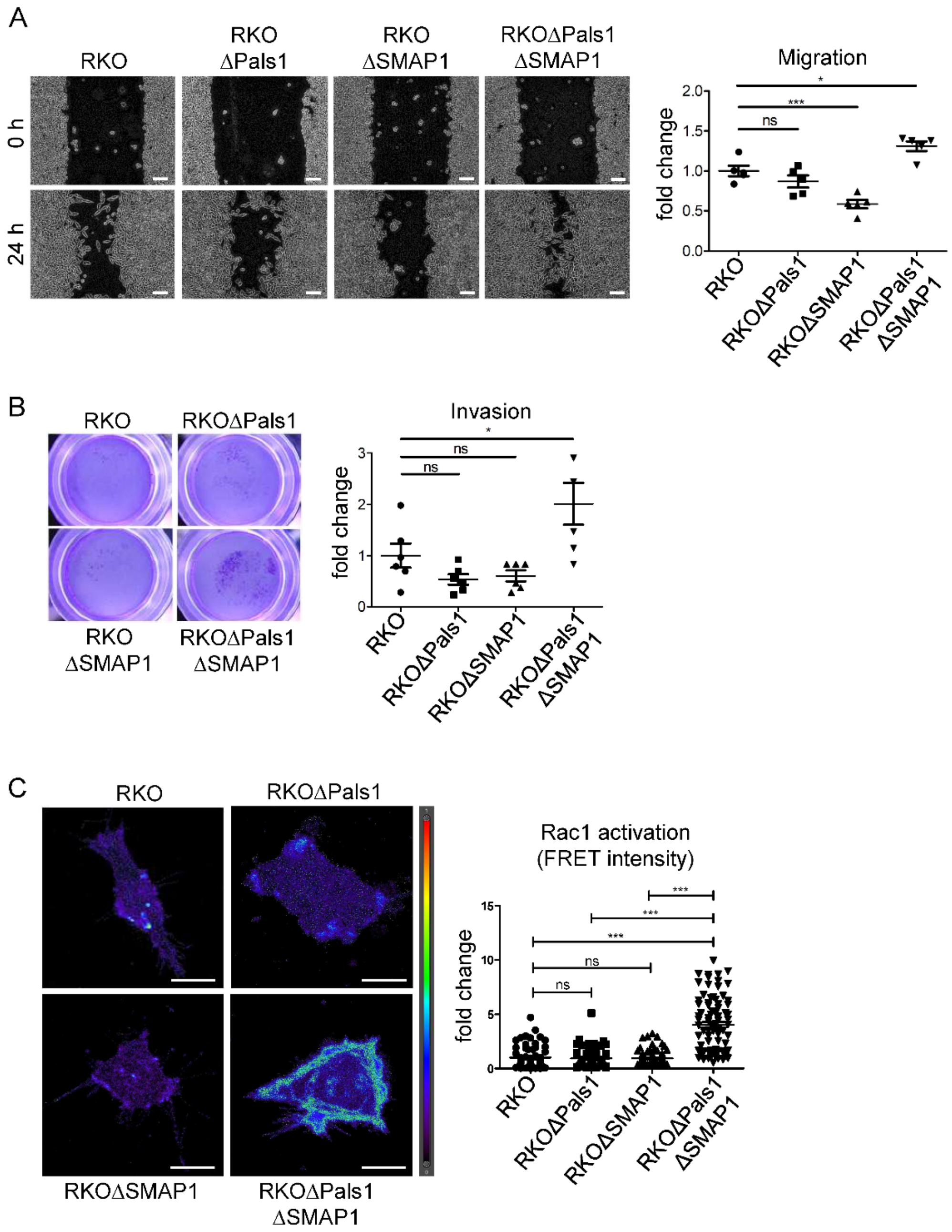

Supplementary Fig. 4: Depletion of SMAP1 and Pals1 results in enhanced cell migration and invasion in RKO cells.

A Representative images from wound healing assays of RKO, RKOΔPals1, RKOΔSMAP1 and RKOΔPals1ΔSMAP1 cells and the corresponding quantification (*N* = 5). **B** Representative images and quantification of transwell matrigel invasion assays of RKO, RKOΔPals1, RKOΔSMAP1 and RKOΔPals1ΔSMAP1 cells (*N* = 6). **C** Representative images and quantification of the FRET signal of a biosensor targeting active Rac1, transfected in the indicated cell lines. Results are representative of 4 experiments. Scale bars are 100µm in B and 20µm in C.

Supplementary Figure 5

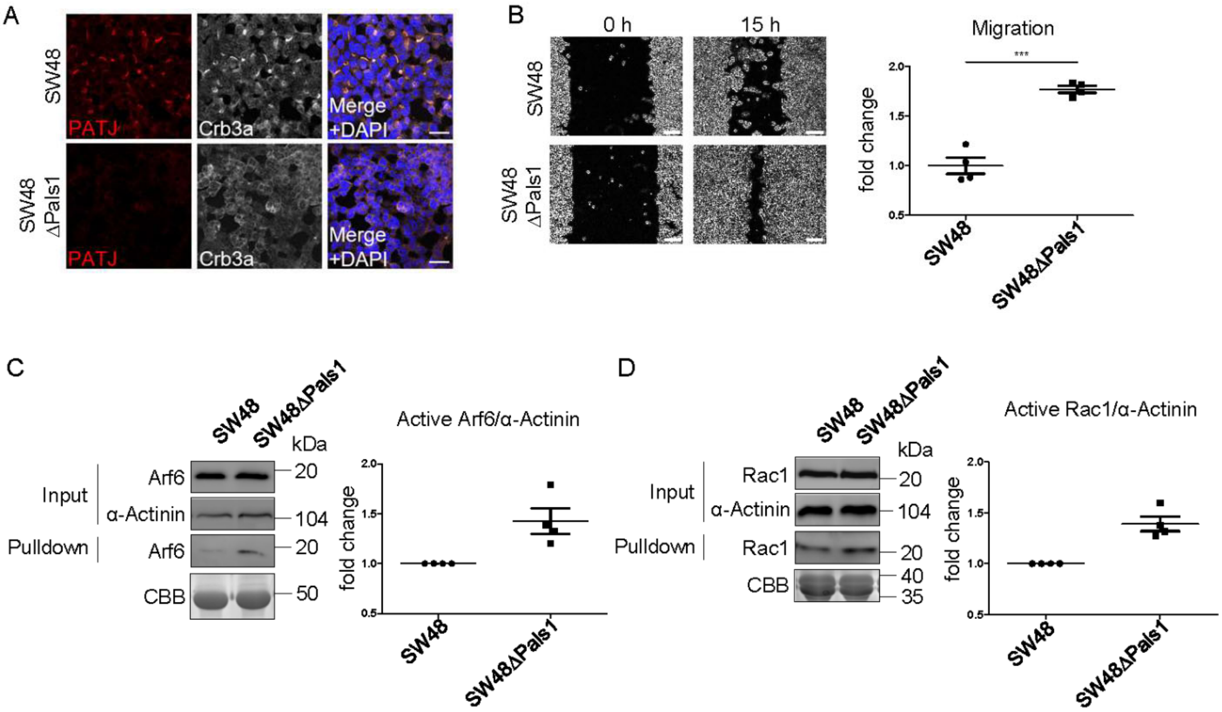

Supplementary Fig. 5: Deletion of Pals1 in SW48 cells results in enhanced cell migration

A Immunostaining of wild type and Pals1-deficient SW48 cell with antibodies against PATJ and Crb3a. **B** Representative images from wound healing assays of SW48 and SW48ΔPals1 cells and the corresponding quantification (N = 4). **C** Western blot and CBB-stained gel of pulldown experiments to detect active Arf6 from cell lysates of SW48 and SW48ΔPals1 cells (N = 6). **D** Western blot and CBB-stained gel of pulldown experiments to detect active Rac1 from cell lysates of SW48 and SW48ΔPals1 cells (N = 3). Scale bars are 20µm in A and 100 µm in B.

Supplementary Fig. 5: Original blots shown in Fig. 1-6.

Figure 2D

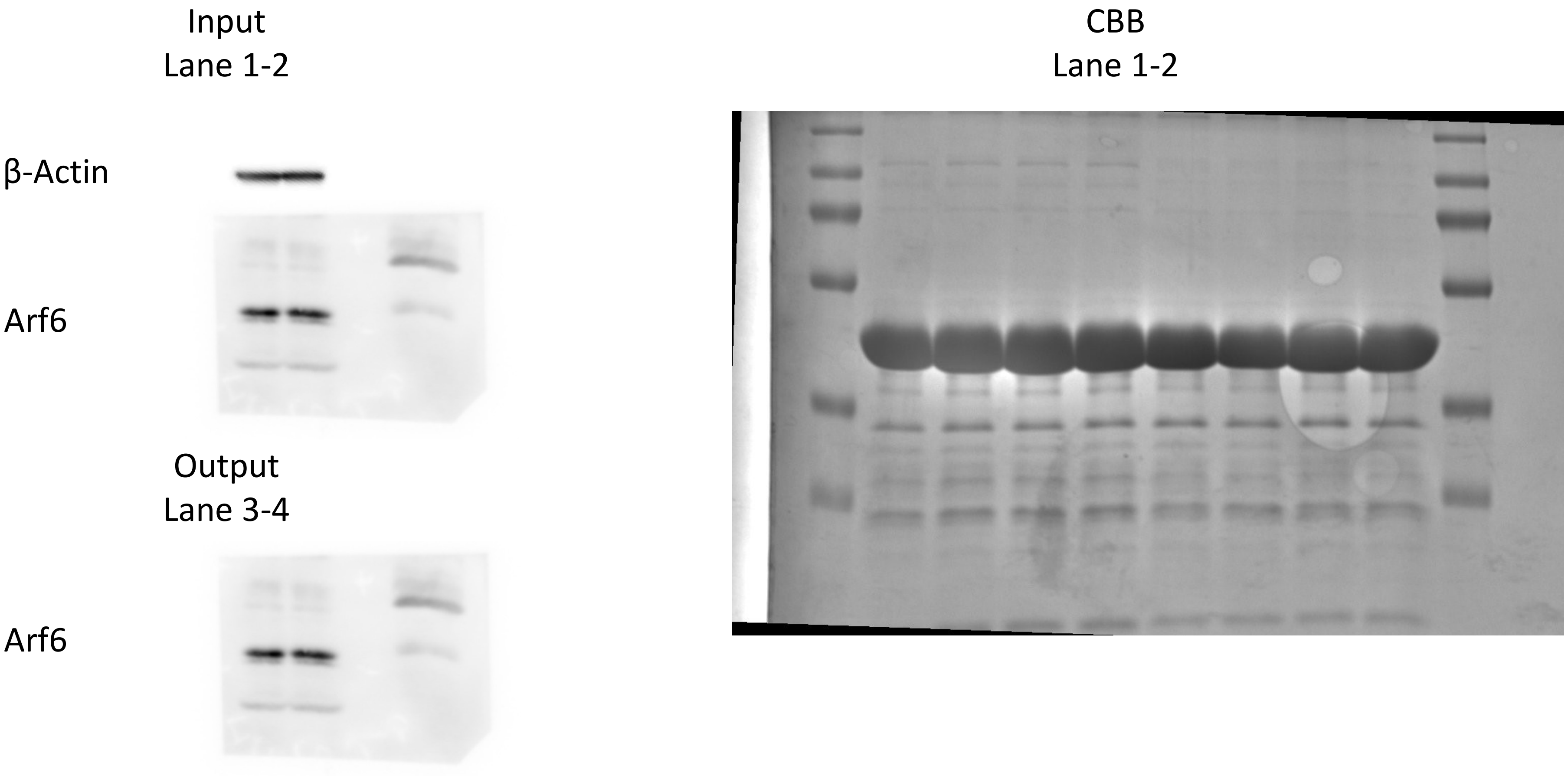

Figure 2E

Input
Lane 7-8

β-Actin

Rac1

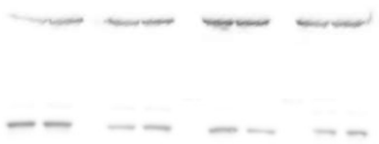

Output
Lane 7-8

Rac1

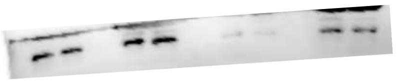

CBB
Lane 7-8

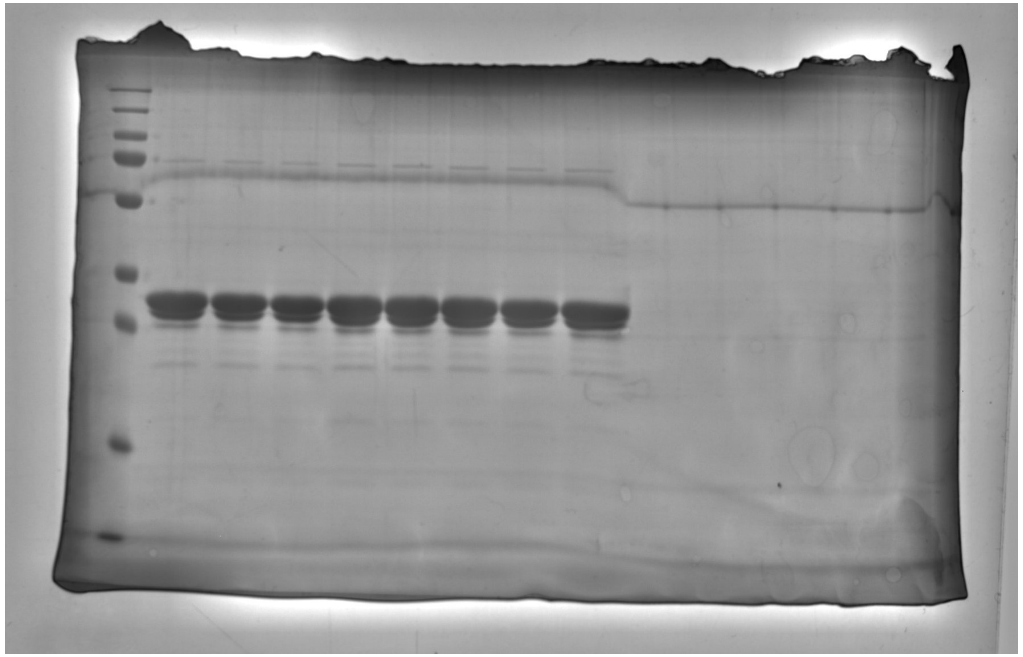

Figure 3D

Input
Lane 3-4

β-Actin

Arf6

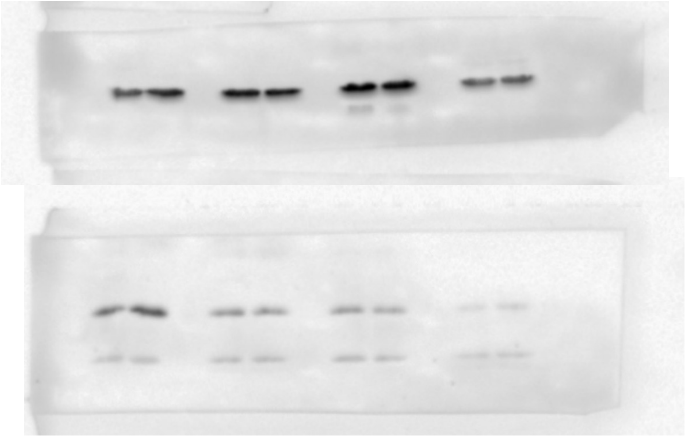

Output
Lane 7-8

Arf6

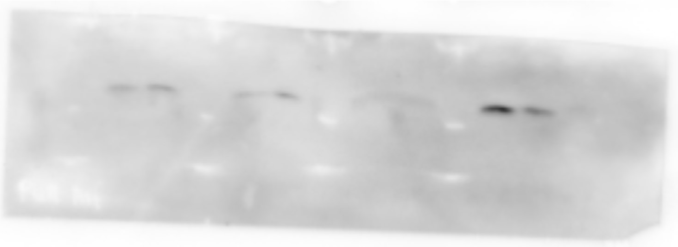

CBB
Lane 1-2

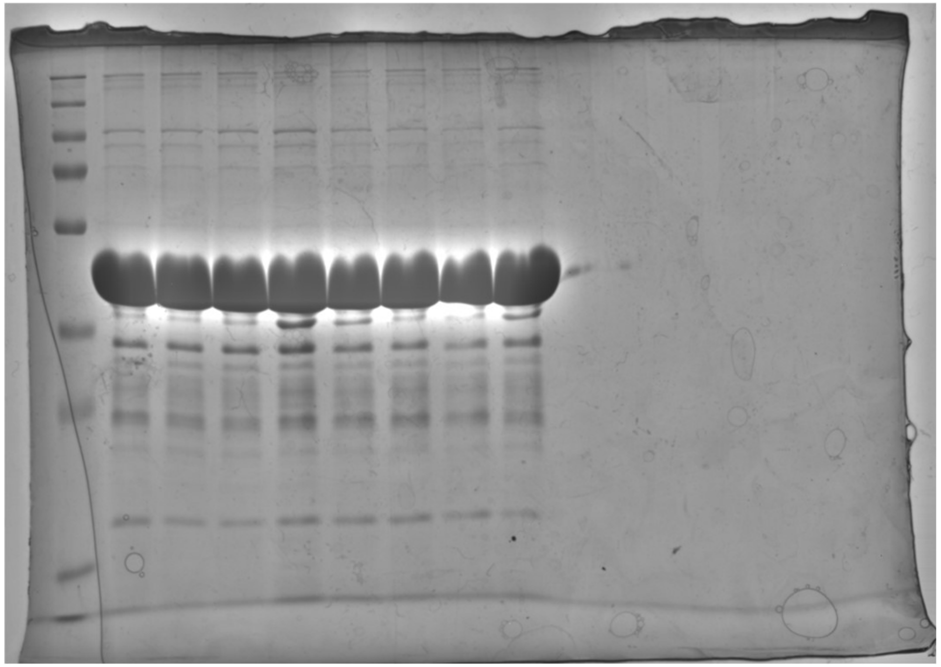

Figure 3E

Input
Lane 3-4

β-Actin

Rac1

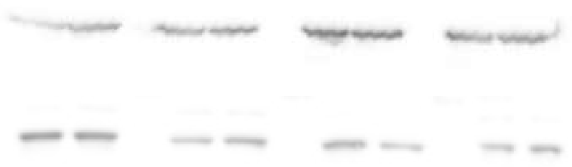

Output
Lane 3-4

Rac1

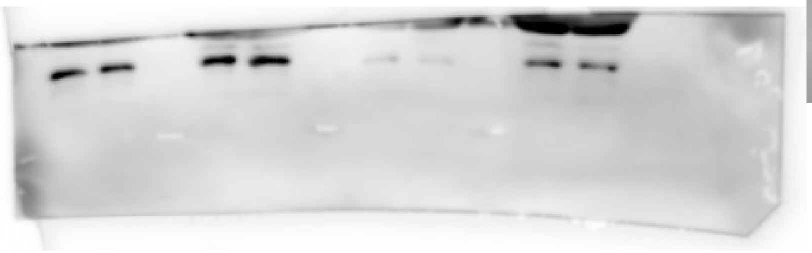

CBB
Lane 3-4

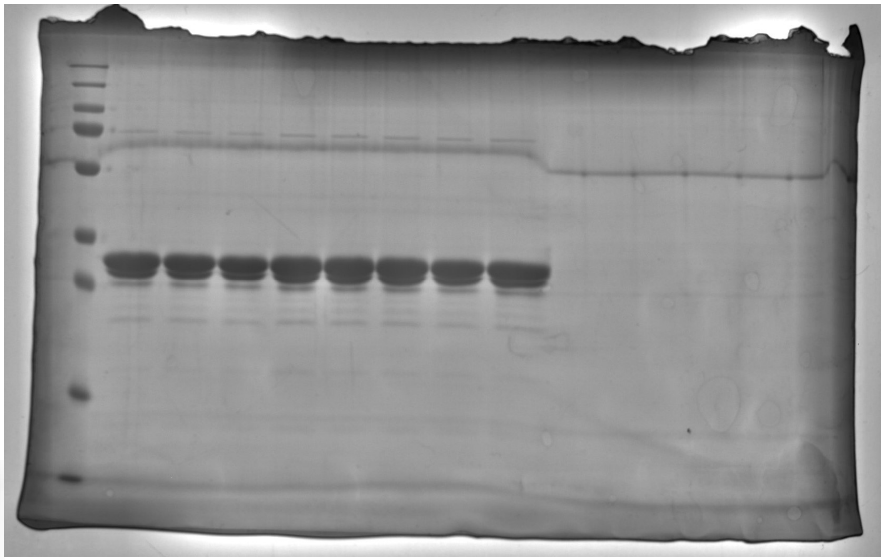

Figure 4A

Lane 1-5

E-Cadherin

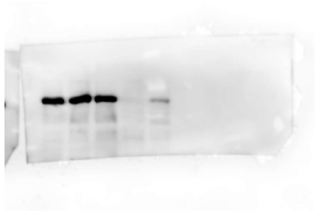

β-Actin

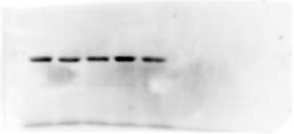

Figure 4D

Input
Lane 1-2

β-Actin

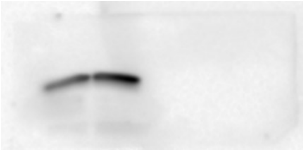

Arf6

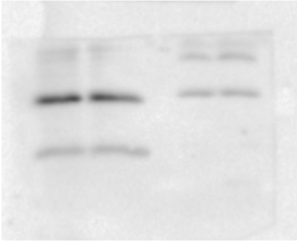

Output
Lane 3-4

Arf6

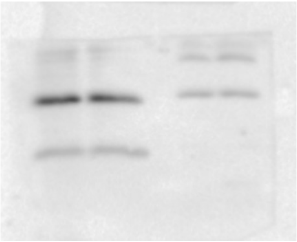

CBB
Lane 3-4

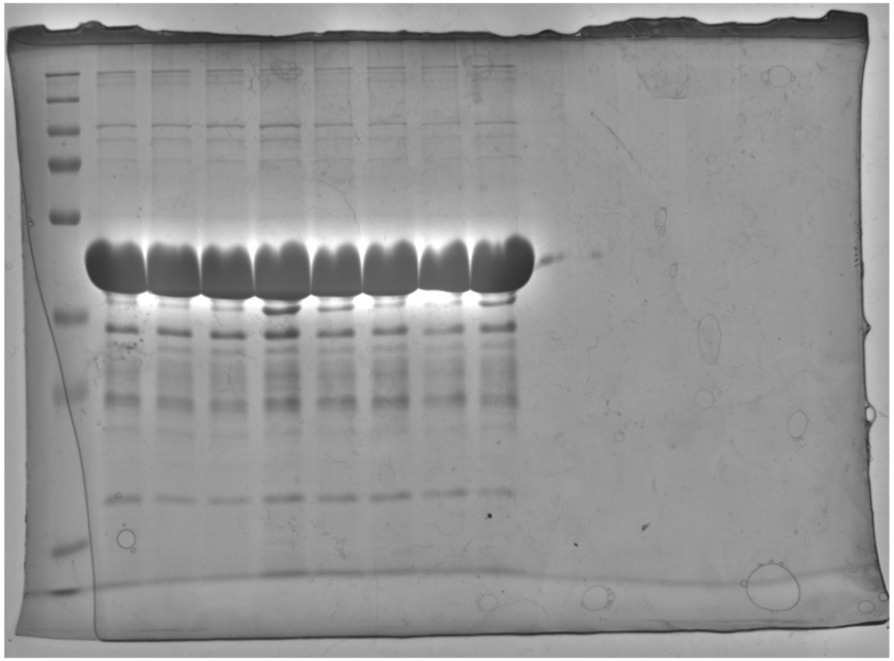

Figure 4E

Input
Lane 5-6

β-Actin

Rac1

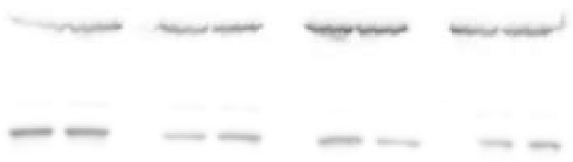

Output
Lane 5-6

Rac1

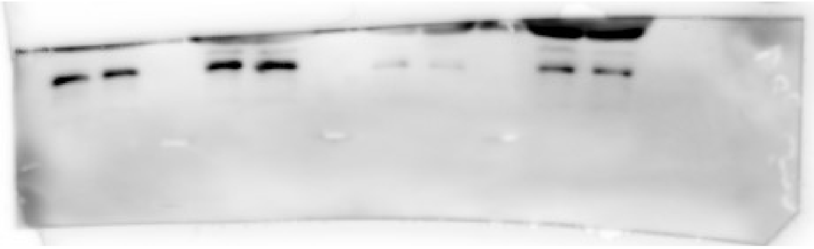

CBB
Lane 5-6

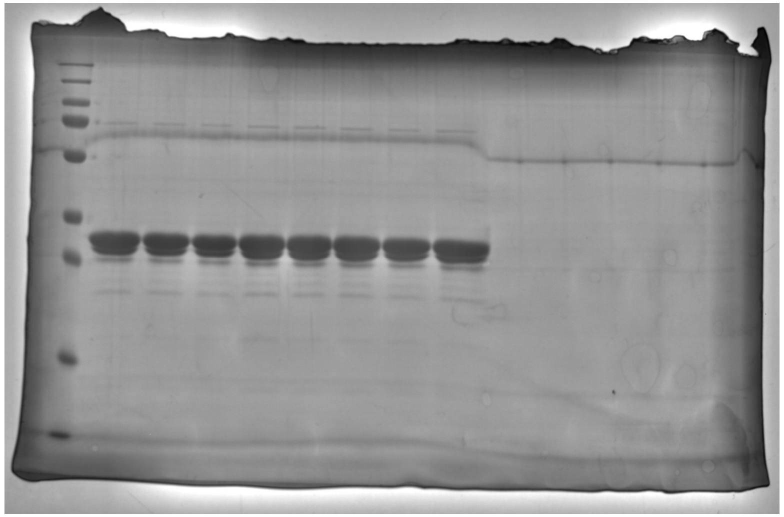

Figure 5A

Lane 1-5

SMAP1

α-Actinin

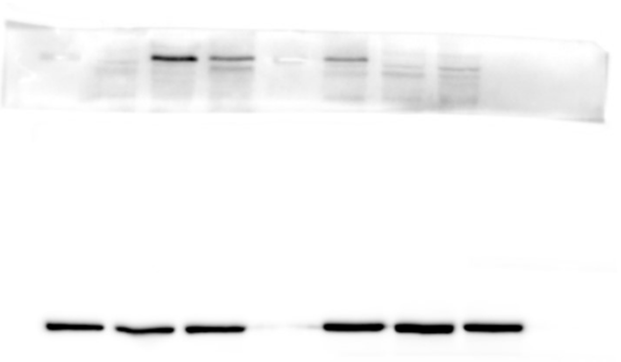

Figure 5D

Input
Lane 1-4

β-Actin

Arf6

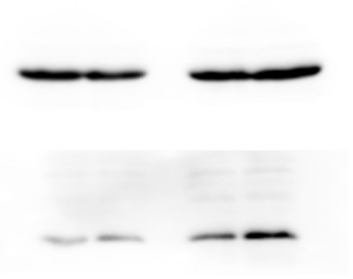

Output
Lane 1-4

Arf6

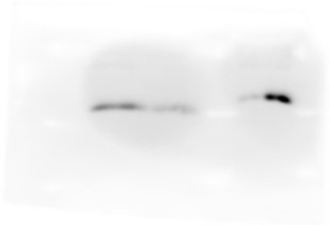

CBB
Lane 1-4

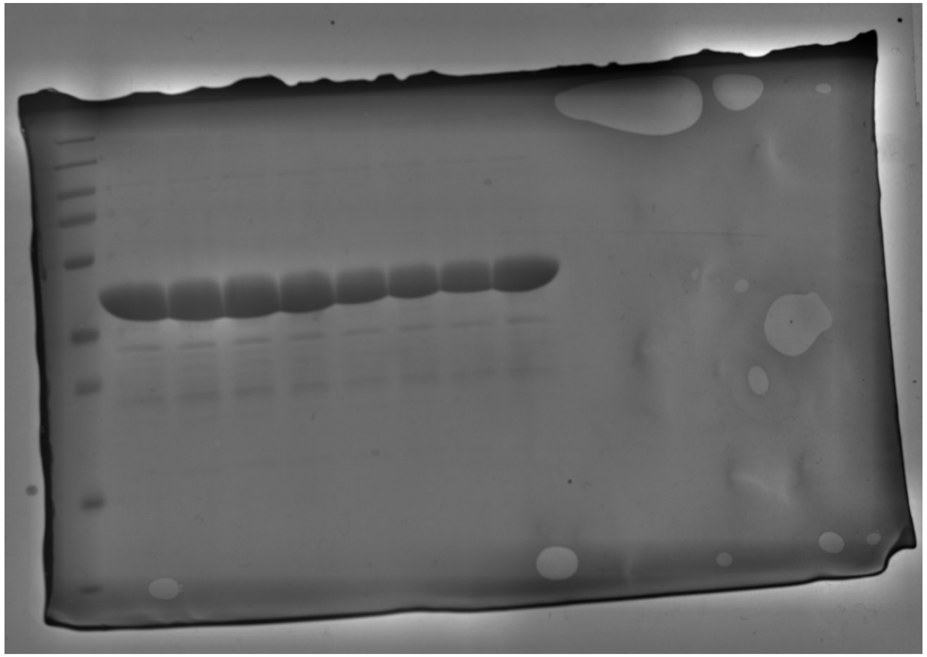

Figure 5E

Input
Lane 1-4

β-Actin

Rac1

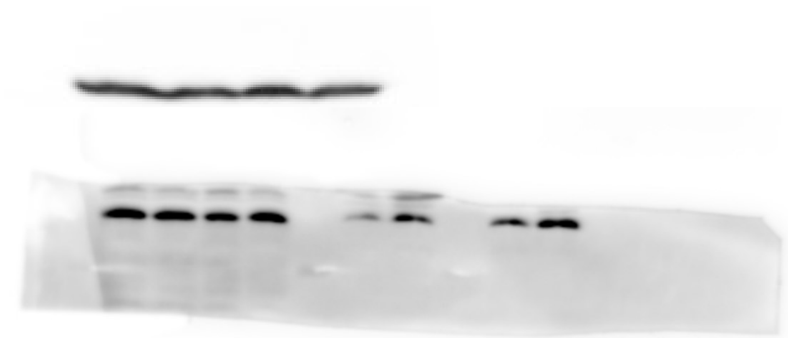

Output
Lane 5-8

Rac1

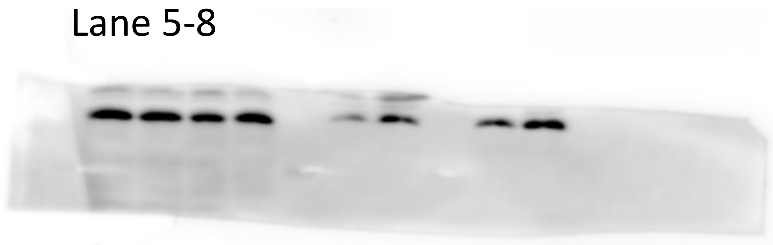

CBB
Lane 5-8

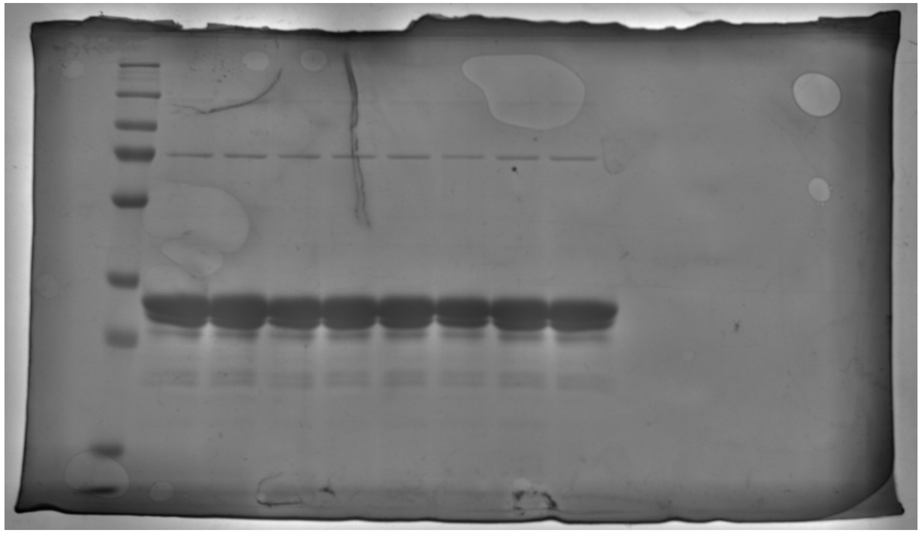

Figure 5F

Lane 1-2

SMAP1

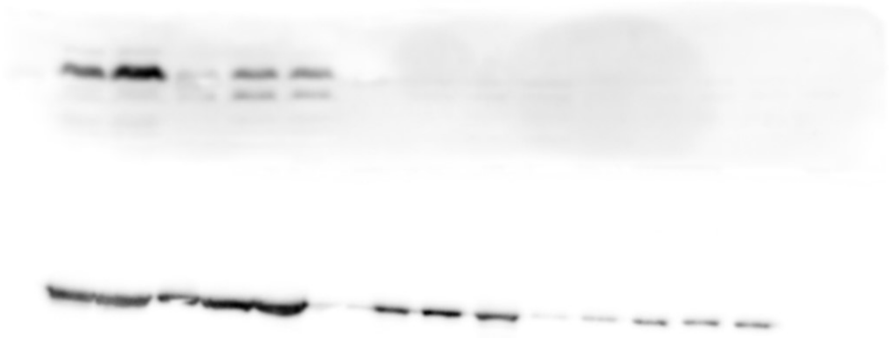

α-Actinin

Figure 5G

Lane 3-4

SMAP1

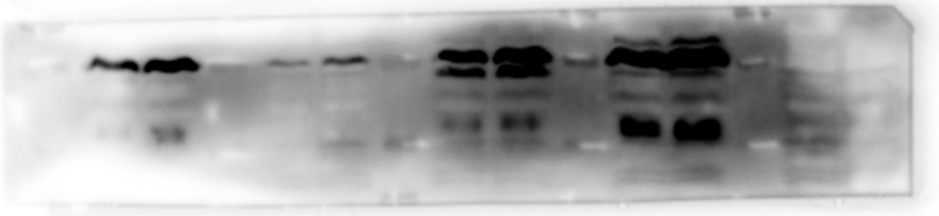

α-Actinin

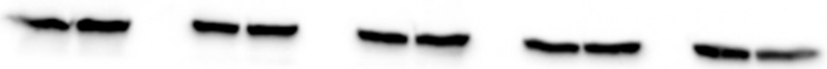

Figure 5H

Lane 5-6

SMAP1

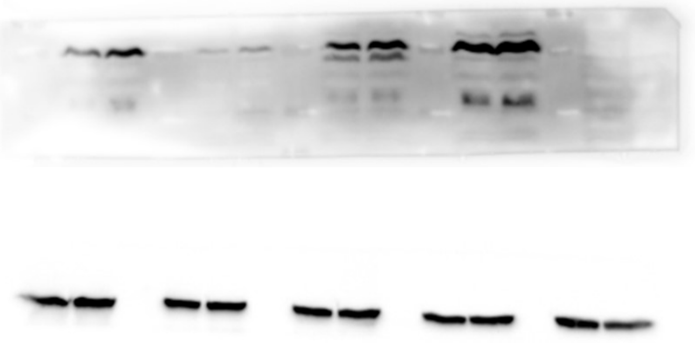

α-Actinin

Figure 6C

Lane 1-6

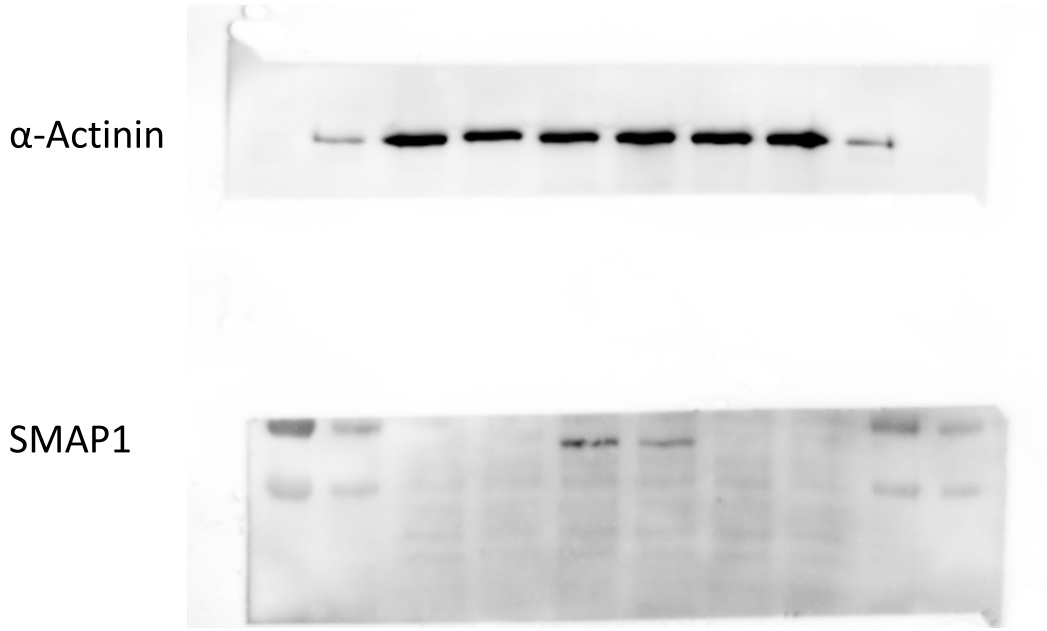

Figure 6G

Input
Lane 1-6

β-Actin

Arf6

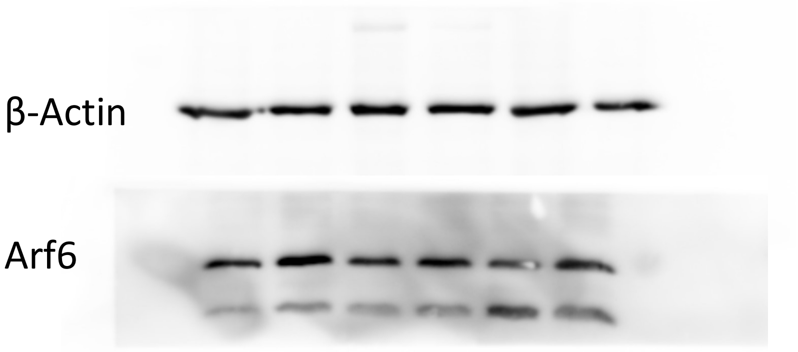

Output
Lane 1-6

Arf6

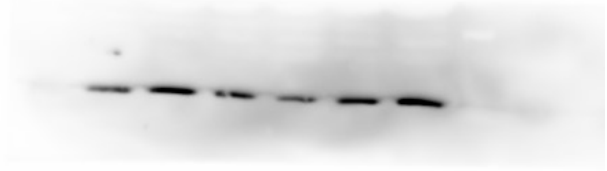

CBB
Lane 1-6

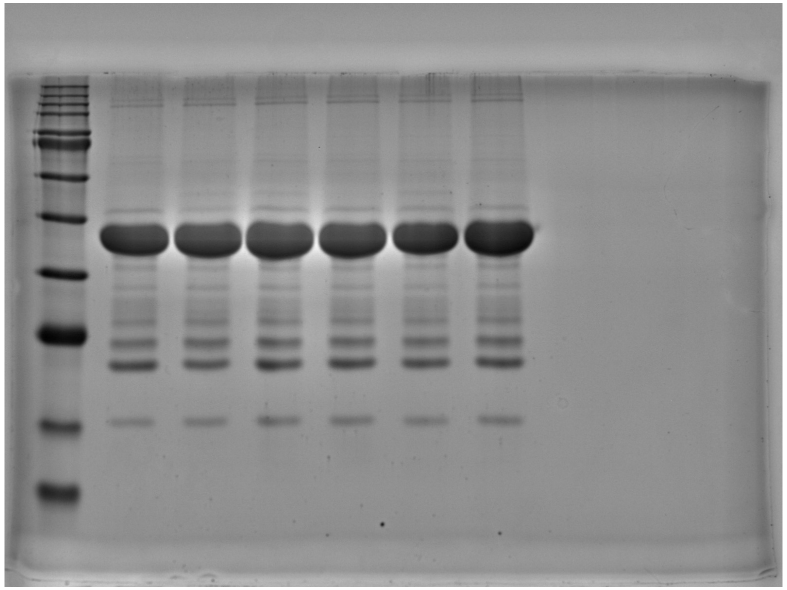

Supplementary Figure 1E

Input
Lane 5-6

α-Actinin

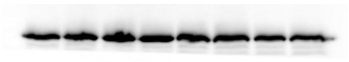

Arf6

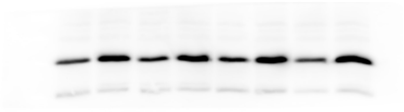

Output
Lane 1-2

Arf6

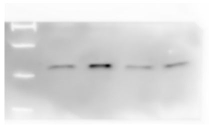

CBB
Lane 5-6

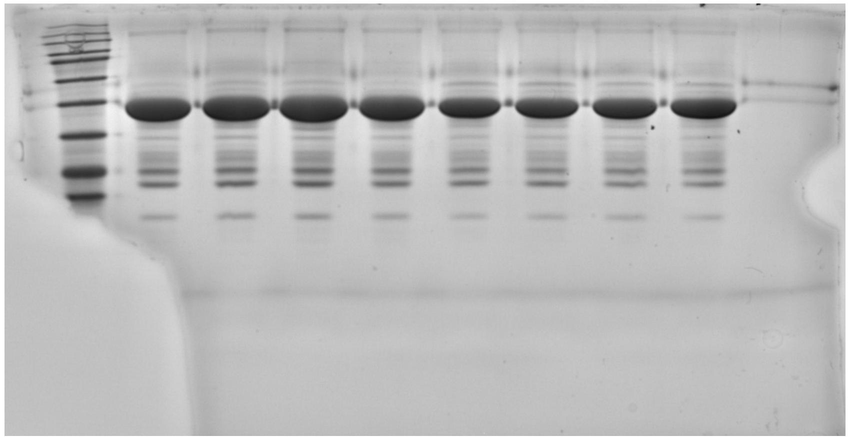

Supplementary Figure 5C

Input
Lane 5-6

α-Actinin

Arf6

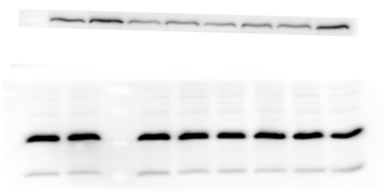

Output
Lane 5-6

Arf6

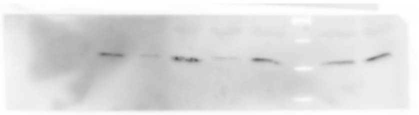

CBB
Lane 5-6

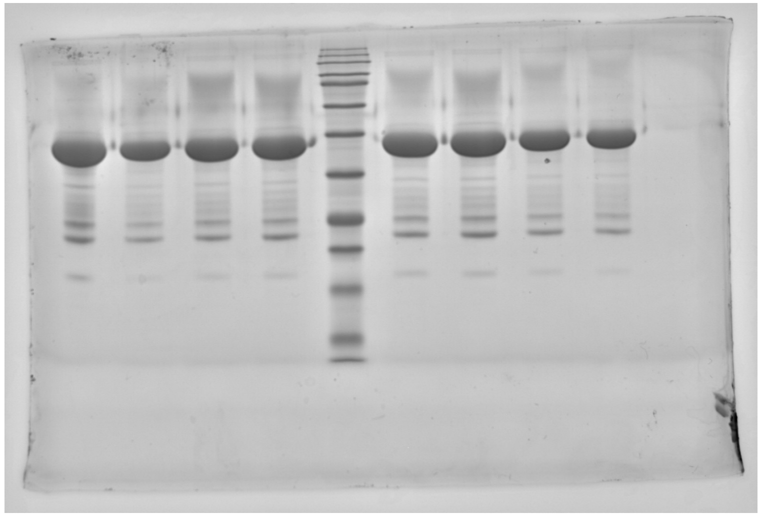

Supplementary Figure 5D

Input
Lane 1-2

α-Actinin

Rac1

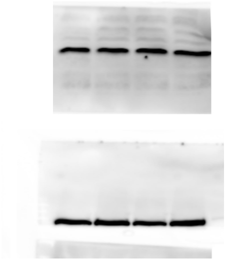

Output
Lane 1-2

Rac1

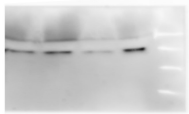

CBB
Lane 5-6

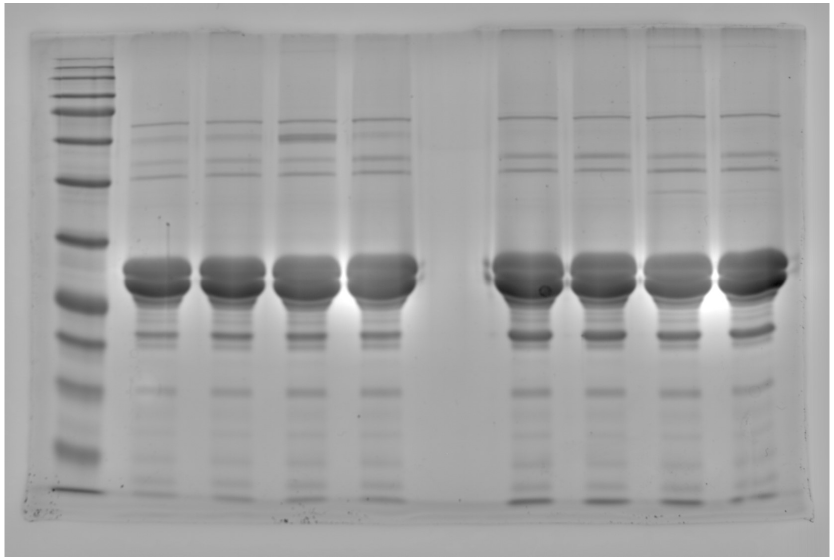

Supplement: Supplementary file 1 — Supplementary Figures 1-6 [file 41417_2022_570_MOESM1_ESM.pdf]
